# A multidisciplinary approach to an unusual medical case of locally advanced gastric cancer: a case report

**DOI:** 10.1186/1752-1947-9-13

**Published:** 2015-01-26

**Authors:** Nicola Carlomagno, Fabrizio Schonauer, Vincenzo Tammaro, Annalena Di Martino, Carmen Criscitiello, Michele L Santangelo

**Affiliations:** 1 General Surgery, Department of Advanced Biomedical Science, University Federico II, Via S. Pansini 5, 80131 Naples, Italy; 2 Plastic and Reconstructive Surgery, Department of Public Health, University Federico II, Via S. Pansini 5, 80131 Naples, Italy; 3 Division of Early Drug Development for Innovative Therapies, European Institute of Oncology, Via Ripamonti 435, 20141 Milan, Italy

**Keywords:** Advanced gastric cancer, Biological prosthesis, Multidisciplinary approach, Neoplastic gastrocutaneous fistula, Reverse abdominoplasty

## Abstract

**Introduction:**

Complete abdominal wall infiltration with neoplastic gastrocutaneous fistula is an unexpected and out of the ordinary presentation of locally advanced gastric cancer. It is very rare to encounter case reports presenting diffuse abdominal wall invasion, but a complete parietal destruction is an exceptional event.

**Case presentation:**

Here we describe the case of an 81-year-old Caucasian woman presenting a carcinoma perforating her anterior gastric wall and infiltrating all layers of her abdominal wall. The gastric tumor infiltrated her transverse mesocolon, the rectus abdominis muscles bilaterally and overran them anteriorly, causing a large parietal deficit and a complete external fistula. Treatment consisted of a complex surgical procedure requiring general and reconstructive surgery cooperation in order to perform an *en bloc* gastric resection including colon and abdominal wall, followed by a parietal reconstruction through positioning of prosthesis and reverse abdominoplasty.

**Conclusions:**

Clinical presentation, histology and therapeutic options are discussed. The importance of a multidisciplinary approach when encountering extremely rare clinical presentations is emphasized.

**Electronic supplementary material:**

The online version of this article (doi:10.1186/1752-1947-9-13) contains supplementary material, which is available to authorized users.

## Introduction

Gastric adenocarcinoma is one of the main causes of cancer-related mortality [1–4]. In some patients diagnosis can be difficult due to various clinical presentations [5–11], some of which are correlated to poor prognosis. Here we report the unusual case of a patient with gastric cancer presenting a large abdominal extramural growth together with a gastrocutaneous fistula.

## Case presentation

**Figure 1:**
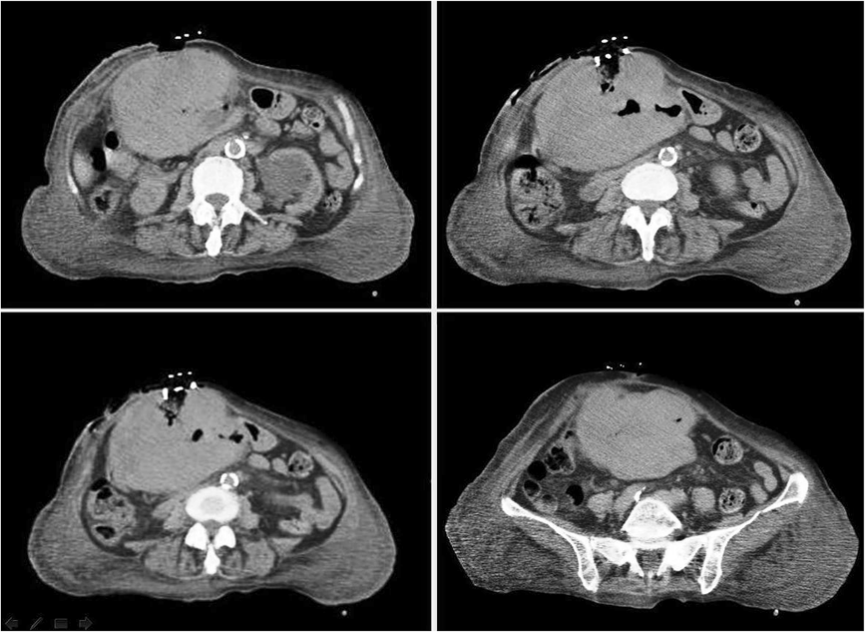
**Computed tomography scan: huge mass arising from the gastric antrum, infiltrating and perforating the abdominal wall.**

**Figure 2:**
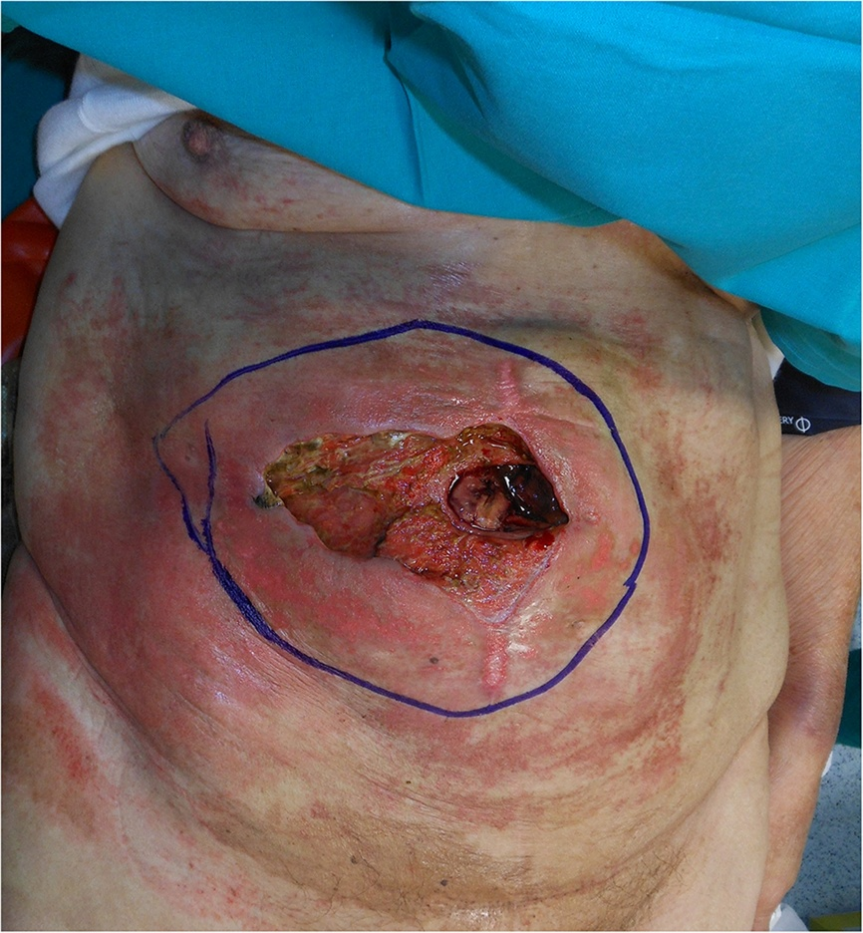
**Preoperative planning: horizontal skin ellipse with 2cm margin of healthy perilesional skin tissue.**

An 81-year-old Caucasian woman with a medical history of hypertension and cholecystectomy for perforated acute cholecystitis was admitted to a community medical ward. At the time, she presented with asthenia, weight loss, occasional vomiting, abdominal pain and anemia. At clinical examination a parietal swelling was observed and at surgical consultation an incisional hernia through the previous cholecystectomy incision was suspected. A gastroscopy revealed the presence of a neoplasm shrinking the gastric antrum. In the following days a small cutaneous ulcer above the parietal swelling was observed followed by the appearance of a fistula a few days later. After surgical consultation she was moved to our university surgical ward.Our laboratory tests revealed hypoproteinemia (4.6g/dL), hypoalbuminemia (2g/dL) and anemia (8.3g/dL) which required transfusion of three blood units. A computed tomography (CT) scan (Figure 1) showed a huge mass (10×14×15cm) arising from her gastric antrum, without a clear cleavage from the left lobe of her liver with internal necrosis and hemorrhage. The mass appeared to infiltrate her transverse mesocolon and her rectus abdominis muscles bilaterally, and to overrun them anteriorly causing a large parietal deficit and a complete external fistula. Furthermore the CT scan did not show large vessel infiltration or distant metastases.At surgery, laparotomy was performed using a skin elliptical incision centered from the neoplastic lesion surrounded by 2cm of healthy perilesional skin tissue (Figure 2). Lateral margins of elliptical incision were enlarged with two other linear incisions.

**Figure 3:**
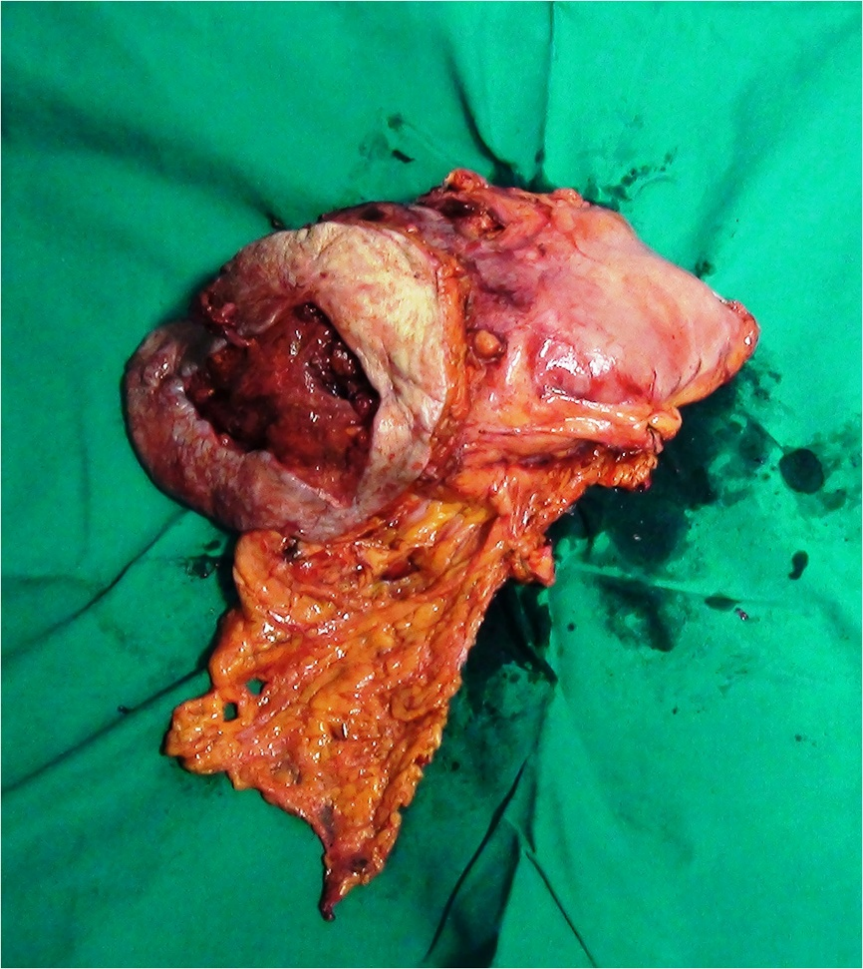
**Specimen of** ***en bloc*** **resection of stomach, transverse colon, rectus abdominis and soft tissue of the abdominal wall.**

**Figure 4:**
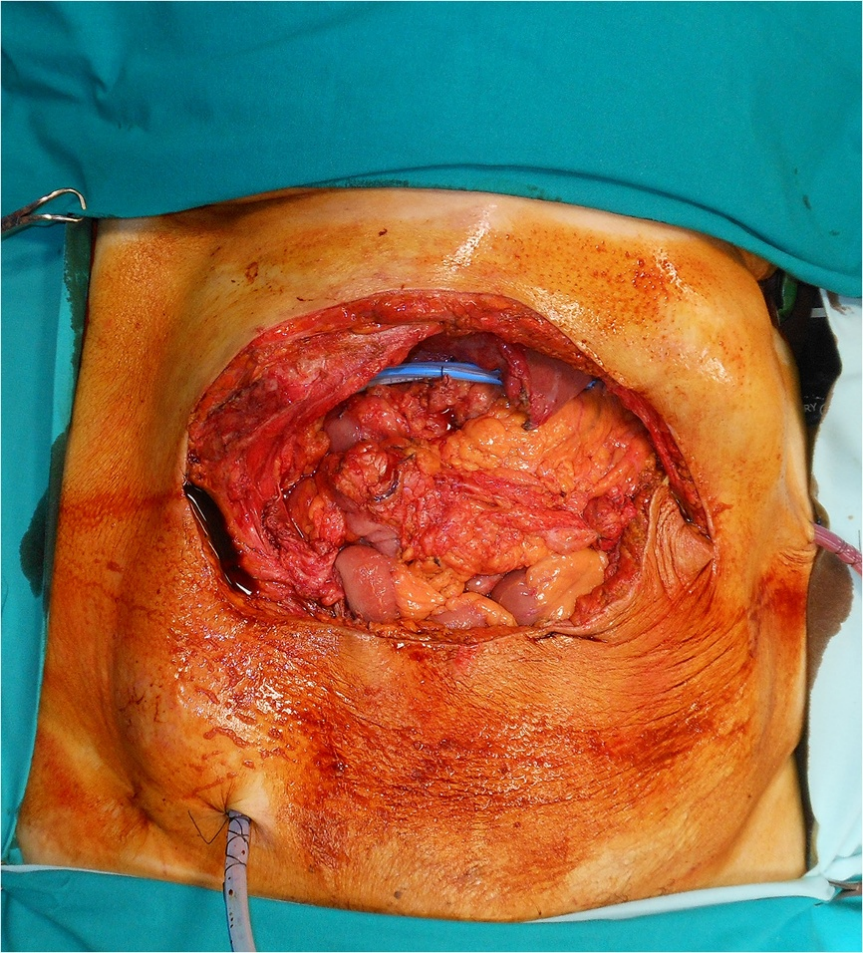
**Residual parietal defect after resection.**

**Figure 5:**
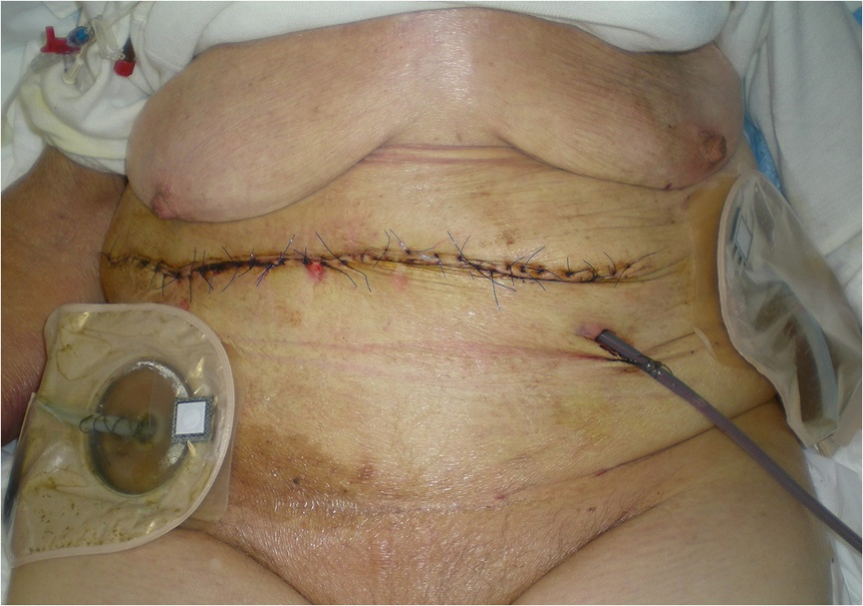
**Skin defect closure with reverse abdominoplasty flap.**

An *en bloc* resection of stomach, colon and abdominal wall was performed (Figure 3) along with systematic lymph node dissection, followed by mechanic gastrojejunostomy and hand-sewn ileocolic anastomosis. Two intra-abdominal drains were positioned and the parietal defect (Figure 4) was reconstructed by positioning a biological prosthesis. The skin defect was closed with reverse abdominoplasty flap [12] (Figure 5). The intra-abdominal pressure (IAP) through intravesical measurement was lower than 10mmHg during the operation and during the postoperative course.

**Figure 6:**
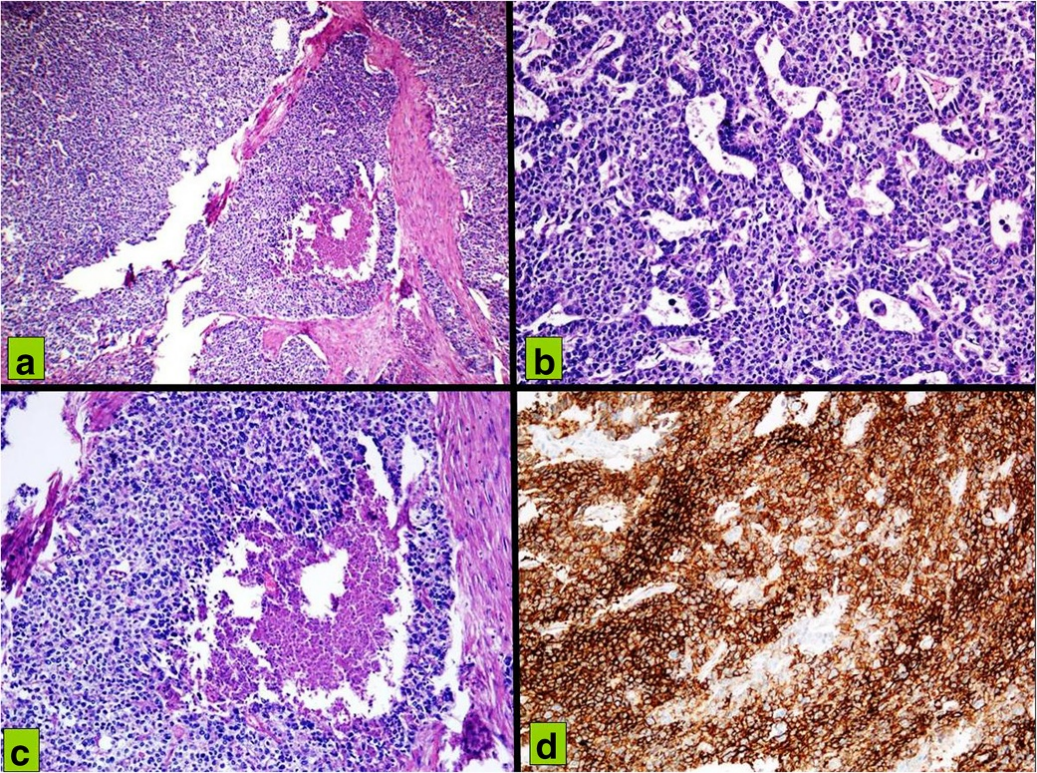
**Hematoxylin and eosin infiltration of gastric wall (a) by cells organized in a solid pattern with foci of necrosis (b) by neoplastic cells with pleomorphic nuclei and high nucleocytoplasmic ratio, with a trabecular and organoid pattern; (c) by tumor cells with vesicular nuclei, amphophilic cytoplasm, in a solid pattern of growth with central necrosis. (d)** At immunohistochemistry neoplastic cells with high CD56 membrane positivity suspicious for neuroendocrine differentiation.

Histology reported a poorly differentiated adenocarcinoma with growth pattern and differentiation phenotype of neuroendocrine type. At microscopy, extension had reached the transverse colon mucosa and the extraserosal tissues up to the skin. (Figure 6a-c). The immunohistochemistry was positive for pan-cytokeratin chromogranin and CD56, (Figure 6d) while negative for vimentin, synaptophysin and S-100.

The immediate postoperative course was satisfactory. The patient did not present relevant laboratory abnormalities except for a mild anemia which was treated with hemotransfusions on the first three postoperative days. On day 3 her bowel movements began. On day 6, after an oral X-ray contrast examination, she started drinking and eating.

Unfortunately, on day 10 she experienced severe respiratory distress that resulted in pneumonia. Despite antibiotic therapy and her transfer to the intensive care unit, she died 20 days after surgery (Additional file 1).

## Conclusions

Although gastric cancer often presents itself at an advanced stage of disease [3, 4], full abdominal wall infiltration along with its destruction and a wide gastrocutaneous fistula is rarely observed. In general, cancer infiltrates abdominal organs by way of diffusion to adjacent tissues and invasion of the superficial serosal layer [5]. Only a few cases of gastric cancer with uncommon clinical presentations have been reported in the literature and these included gastrointestinal obstruction [6–9], proctorrhagia, straining and rectal tenesmus [10]. To the best of our knowledge, our patient’s gastric cancer presentation as extramural diffusion and abdominal wall fistula is a very rare occurrence. Waguri *et al.*[11] reported the case of a 62-year-old man affected by gastric cancer and treated with a weekly low-dose paclitaxel who presented with diffuse abdominal wall invasion, but without a complete parietal destruction.

Recent reports of gastric tumors characterized by extremely large dimensions or rapid growth have often revealed – at histology – gastrointestinal stromal tumors (GISTs) [13, 14] rather than adenocarcinoma. Of interest, none of the above revealed GISTS of the neuroendocrine type as in our reported case.

In advanced gastric cancer possible therapeutic options include neoadjuvant chemotherapy with gastrostomy or jejunostomy. However, although evaluated by a multidisciplinary team, such a standard approach would not have been feasible in our case due to the fact that the huge parietal defect, the perforation, the stenosis and, above all, the transverse colon involvement represented a contraindication to performing either a gastrostomy or a jejunostomy. The massive cancer infiltration of the wall and surrounding organs, without great vessels infiltrations, led us to perform a gastric resection including transverse colon, rectus abdominis muscles and skin.

The large parietal defect was reconstructed by positioning a biological prosthesis which is the gold standard procedure when tissue contamination is present or high risk of anastomotic dehiscence exists [15].

Full-thickness abdominal defects following oncological resection are considered a reconstructive challenge, especially when wide excision margins are required to avoid cancer recurrence. Most advantageous reconstructive options may involve local, pedicled or free flap transfer [3]. Local flaps may be either not available due to the disease itself or jeopardized due to radiotherapy.

Pedicled superior or inferior rectus abdominis flap or extended latissimus dorsi flap can be used for upper abdominal wall reconstruction. In some cases (that is, severely damaged local tissues), free tissue transfer may be required for reconstruction [16]. Although free flaps have some advantages as compared to pedicled flaps such as more reliable healing, they also have some disadvantages including longer operative time, possible total flap failure and distant donor site morbidity.

Reverse abdominoplasty may represent a valuable alternative to pedicled or free flap reconstruction due to its recruitment of the adjacent abdominal tissue into the defect [17]. The inferior resection margin of the tumor ablation represents the upper border of the flap, raised below Scarpa’s fascia, above the rectus sheath. The dissection then continues to the pubic area and the inferior abdominal flap is then advanced cranially to cover the upper abdominal wall defect. Despite its potential as a reconstructive procedure, reverse abdominoplasty is usually performed in post-massive weight loss surgery [18, 19] and rarely for reconstruction following oncological resection [12, 20, 21]. Reverse abdominoplasty can be used in combination with other flaps, or as soft tissue coverage over a prosthetic mesh.

Based on our experience, we can therefore consider reverse abdominoplasty a valuable surgical option when confronting extraordinary parietal destruction as it enables the surgeon to create a skin cover for an abdominal wall defect. Considering that our patient was elderly with skin laxity, we were able to obtain a large inferior abdominal flap by employing this technique.

The above described clinical presentation of gastric cancer is quite rare and even rarer in the Western hemisphere. Proper management of gastric cancer requires a multidisciplinary approach involving the oncologist, surgeon and radiation oncologist. This is particularly paramount when gastric cancer has an unfamiliar manifestation such as in our reported case. When such rare cases are presented, an even larger pool of specialists has to be involved in the management of the disease. In our experience we benefited from involving a plastic surgeon in our integrated approach to patient treatment.

Although the reported case had an unsatisfactory outcome and adversely ended with the death of the patient, it is worth highlighting that this was not due to surgical complication. The measurement of IAP has been incorporated routinely in critical units in order to monitor and control those clinical situations that make us suspicious of intra-abdominal hypertension (IAH). Abdominal compartment syndrome (ACS) and IAH are increasingly recognized as potential complications in patients in intensive care units. ACS and IAH affect all body systems, most notably the cardiac, respiratory, renal, and neurologic systems. ACS/IAH affects blood flow to various organs and plays a significant role in the prognosis of the patients. Recognition of ACS/IAH, its risk factors and clinical signs can reduce the morbidity and mortality associated [22]. We used the intravesical measurement, the most common method. In our case IAP was always normal at intraoperative measurement and during postoperative course. Indeed, as the planned and performed surgery was the result of a collaboration between the general and plastic surgeon, beyond focusing on the excisional components of the procedure (*en bloc* resection of the tumor burden including stomach/colon/abdominal wall), our approach was to frame the reconstructive issue, which was carried out by implementing adequate surgical techniques and devices. In conclusion, as our case study attests, we are convinced of the utility and importance of a multidisciplinary approach when confronted with highly abnormal cancerous growths. We would like to cite the additional file at the end of the case presentation as summary of the all the clinical history.

## Consent

Written informed consent was obtained from the patient’s next-of-kin for publication of this case report and any accompanying images. A copy of the written consent is available for review by the Editor-in-Chief of this journal.

## Electronic supplementary material

Additional file 1: **Absence of genetic history; absence of environmental and lifestyle influences.** (DOC 36 KB)
