# Supplementary material for: A multidisciplinary approach to an unusual medical case of locally advanced gastric cancer: a case report
Source: J Med Case Rep. 2015 Jan 26;9:13. doi: 10.1186/1752-1947-9-13 (PMC4407615; doi:10.1186/1752-1947-9-13)
Supplement: Supplementary file 1 — Additional file 1: Absence of genetic history; absence of environmental and lifestyle influences. (DOC 36 KB) [file 13256_2014_3092_MOESM1_ESM.doc]

Laboratory: hypoproteinemia (4.6g/dL), hypoalbuminemia (2g/dL) and anemia (8.3g/dL)

First admission at a medical ward

On day 10 severe respiratory distress that resulted in pneumonia.

New transfer to intensive care

Past illness: perforated acute cholecystitis biliary lithiasis, hypertension, frequent urinary infections

Transfusion of three blood units, hydro-electrolyte balance

History: asthenia, weight loss, abdominal pain, anemia, and

parietal swelling considered as an incisional hernia

Gastroscopy: vegetating neoplasm stenosing the gastric antrum

Antibiotic therapy, patient transfer to intensive care unit on days 0–3

**Absence**

**of genetic Birth in 1931 Past history Presenting concerns Diagnosis Intervention Postoperative course**

**history;**

**absence of**

Over 40 years ago:

laparotomy –cholecystectomy

and main biliary duct exploration

Neoplastic lesion, involving gastric body and antrum, determining protrusion and perforation of the anterior gastric wall with infiltration and destruction of the abdominal wall

**Computed tomography scan:**  huge mass arising from the gastric antrum, without a clear cleavage from the left lobe of liver, with internal necrosis and hemorrhage.

Infiltration of the transverse mesocolon, the rectus abdominis muscles with a large parietal deficit and a complete external fistula without large vessels infiltration or distant metastases

**environmental**

An ***en bloc* resection** of stomach, colon and abdominal wall was performed along with systematic lymph node dissection, mechanic gastrojejunostomy and hand-sewn ileocolic anastomosis

positioning a **biological prosthesis.**

Closure of skin defect with **reverse abdominoplasty flap**

Death on day 20

**and**

**lifestyle**

**influences**

Second admission at our surgical ward

**Histology:** poorly differentiated adenocarcinoma with growth pattern and differentiation phenotype of neuroendocrine type.

The immunohistochemistry was positive for pan-cytokeratin, chromogranin and CD56 while negative for vimentin, synaptophysin and S-100
